# miR-135A Regulates Preimplantation Embryo Development through Down-Regulation of E3 Ubiquitin Ligase Seven in Absentia Homolog 1A (SIAH1A) Expression

**DOI:** 10.1371/journal.pone.0027878

**Published:** 2011-11-22

**Authors:** Ronald T. K. Pang, Wei-Min Liu, Carmen O. N. Leung, Tian-Min Ye, Peter C. K. Kwan, Kai-Fai Lee, William S. B. Yeung

**Affiliations:** 1 Department of Obstetrics and Gynaecology, The University of Hong Kong, Pokfulam, Hong Kong, People's Republic of China; 2 Centre for Reproduction, Development and Growth, The University of Hong Kong, Pokfulam, Hong Kong, People's Republic of China; State Key Laboratory of Reproductive Biology, Institute of Zoology, Chinese Academy of Sciences, China

## Abstract

**Background:**

MicroRNAs (miRNAs) are small non-coding RNA molecules capable of regulating transcription and translation. Previously, a cluster of miRNAs that are specifically expressed in mouse zygotes but not in oocytes or other preimplantation stages embryos are identified by multiplex real-time polymerase chain reaction-based miRNA profiling. The functional role of one of these zygote-specific miRNAs, miR-135a, in preimplantation embryo development was investigated.

**Methodology/Principal Findings:**

Microinjection of miR-135a inhibitor suppressed first cell cleavage in more than 30% of the zygotes. Bioinformatics analysis identified E3 Ubiquitin Ligase Seven In Absentia Homolog 1A (Siah1a) as a predicted target of miR-135a. Western blotting and 3′UTR luciferase functional assays demonstrated that miR-135a down-regulated the expression of Siah1 in HeLa cells and in mouse zygotes. Siah1a was expressed in preimplantation embryos and its expression pattern negatively correlated with that of miR-135a. Co-injection of Siah1a-specific antibody with miR-135a inhibitor partially nullified the effect of miR-135a inhibition. Proteasome inhibition by MG-132 revealed that miR-135a regulated proteasomal degradation and potentially controlled the expression of chemokinesin DNA binding protein (Kid).

**Conclusions/Significance:**

The present study demonstrated for the first time that zygotic specific miRNA modulates the first cell cleavage through regulating expression of Siah1a.

## Introduction

Mature microRNAs (miRNAs) are endogenous non-coding, small RNAs that regulate gene expression through mRNA degradation or translation suppression by complementary pairing to the 3′-untranslated region (3′-UTR) of specific target mRNAs [1]–[3]. MiRNAs are involved in various biological processes; however, their role in preimplantation embryo development is controversial. Mouse oocytes without a miRNA-processing enzyme termed dicer do not have miRNAs and exhibit disorganized spindle [4]. Embryos deriving from these dicer deficient oocytes cannot pass through the first cleavage [4]. Evidence also indicates that miRNAs control a proportion of maternal genes in the mouse preimplantation embryos [4]. In zebrafish zygotes, miR-430 is essential for facilitating the deadenylation and clearance of maternal mRNAs [3]. These data, together with the functional studies in *C. elegans* [5]–[7], *Drosophila* [8]–[10] and fish [11], supports the belief that miRNAs are critical in key developmental events in vertebrates and invertebrates [12], [13]. However, mouse blastocysts deficient of Dgcr8, another major miRNA processing enzyme, appear to be normal [14], [15], though they die at E6.5 [16]. Other contradictory reports show that miRNA function is suppressed in oocytes [14], [15].

Protein degradation via the ubiquitin- proteasome pathway is essential in diverse aspects of normal cell physiology and development. In the early embryo; protein degradation is vital in the transition from maternal to embryonic control of development [17], [18]. The transition involves not only production of new mRNAs but also protein turnover. Ubiquitination targeted protein proteolysis in eukaryotic cells [19] and is important in cell cycle progression [20], [21]. Disruption of the ubiquitin-proteasome pathway affects normal embryo development. In early Xenopus embryo, destruction of cytoplasmic polyadenylation element binding protein (CPEB) is necessary for mitosis to take place [20], [21].

There are 3 types of enzymes involving in ubiquitination, namely E1, E2 and E3. E1 are ubiquitin-activating enzymes. They form a thiol-ester linkage with ubiquitin, which is then transferred to the E2 ubiquitin-conjugating enzyme. The E3 enzymes are protein ligases that transfer ubiquitin from the E2 enzyme to the lysine residues of specific proteins, thus targeting them for degradation by the proteasome. The E3 ubiquitin ligases control the specificity of ubiquitination by interacting only with specific target proteins. The E3 ubiquitin ligases contain a signature RING (Really Interesting Novel Gene) finger consensus sequence. Majority of the RING finger-containing proteins function as ubiquitin protein ligase [22], [23].

Seven in absentia homolog 1 (Siah1) is a member of the RING finger proteins with E3 ligase activity. It is a highly related mammalian homolog of the Drosophila SINA, that has been implicated in the ubiquitination and proteasome-dependent degradation of various molecules [24], [25]. There are two Siah genes in humans, Siah1 and Siah2 [25] while there are two Siah1 genes (Siah1a and Siah1b) and a single Siah2 gene in mice [24]. Siah1 expression is induced by p53 in mammals and over-expression of Siah1 inhibits cell proliferation and promotes apoptosis [26]–[28]. Siah1 is also important for early embryo development; the birth frequency of Siah1 hemizygous (null) mice is very low and many of their embryos exhibit developmental abnormalities [29].

Our previous miRNA profiling of mouse spermatozoa, oocytes and preimplantation embryos indicated that miR-135a was highly expressed in zygotes, but decreased gradually with development (). This study aimed to confirm this observation and to study the function of miR-135a in the zygotes. Here, we provide evidence that miR-135a regulates the first cell division mediated by suppressing the expression of Siah1a, which in turns affect destabilization of chemokinesin DNA binding protein (Kid), which mediates chromosome compaction and is degraded by the proteasome pathway during mitosis [30], [31].

## Results

### Expression of miR-135a in preimplantation embryos

**Figure 1:**
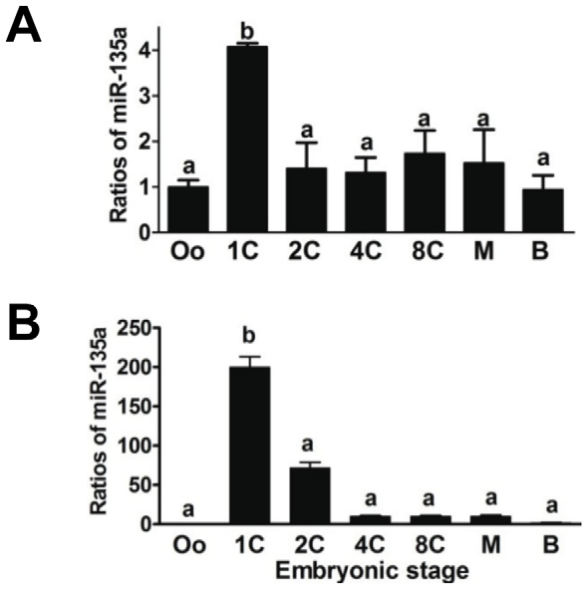
MiR-135a expression in preimplantation mouse embryos. The expressions of miR-135a at different developmental stages were assessed by qPCRs with (A) and without preamplification (B) on 5 embryos (n = 5). The ratios were calculated against Ct values of oocytes and normalized by endogenous RNA U6 expression. Different developmental stages were denoted as oocytes (Oo), zygotes (1C), 2-cell (2C), 4-cell (4C), 8-cell (8C), morula (M) and blastocyst (B). ^a–b^ indicates significant difference between groups (*p*<0.05).

Our previous miRNA profiling showed that miR-135a was highly expressed in the zygotes and decreased thereafter (Figure 1A). The observation was confirmed by independent real-time qPCR. We found that miR-135a was expressed in all preimplantation embryos but the level was significantly higher at the zygote stage than at other stages of development (Figure 1B). This stage-specific expression of miR-135a prompted us to investigate its functional roles in zygotic development.

### MiR-135a inhibition suppresses first cell cleavage

**Figure 2:**
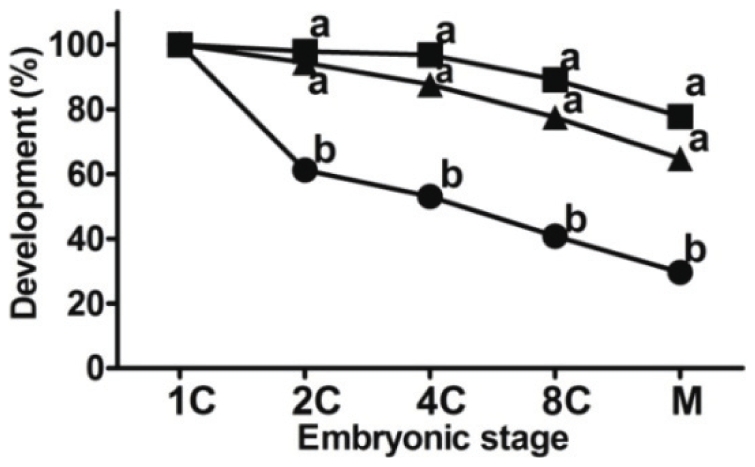
Effects of miR-135a inhibition on the development of mouse preimplantation embryos. Microinjection of miR-135a inhibitor suppressed the development of 1-cell embryo. Development percentage was based on number of 1-cell embryos used. Statistical significant differences were found between the miR-135a-injected embryos (circle) and the scramble control-injected embryos (triangle) and the untreated control (square). The experiment was repeated for at least 3 times, and at least 100 embryos were involved in each treatment. Different embryo developmental stages were denoted as zygotes (1C), 2-cell (2C), 4-cell (4C), 8-cell (8C) and morula (M). ^a–b^ denotes significant difference (*p*<0.05) between treatments at the same time point.

We hypothesized that miR-135a affected first zygotic cleavage. The hypothesis was tested by microinjection of miR-135a inhibitor into the pronucleated zygotes at 21-hour post-human chorionic gonadotrophin (hCG) administration. MiR-135a knockdown significantly reduced cell division of zygotes when compared to those injected with scramble control; more than 30% of the miR-135a inhibitor-treated zygotes failed to advance to the 2-cell stage while more than 94% of the scramble control-injected zygotes developed to the 2-cell embryo (Figure 2). There was no significant difference in the development of untreated embryos (without injection) and those injected with scramble control.

### MiR-135a regulates the expression of Siah1

**Figure 3:**
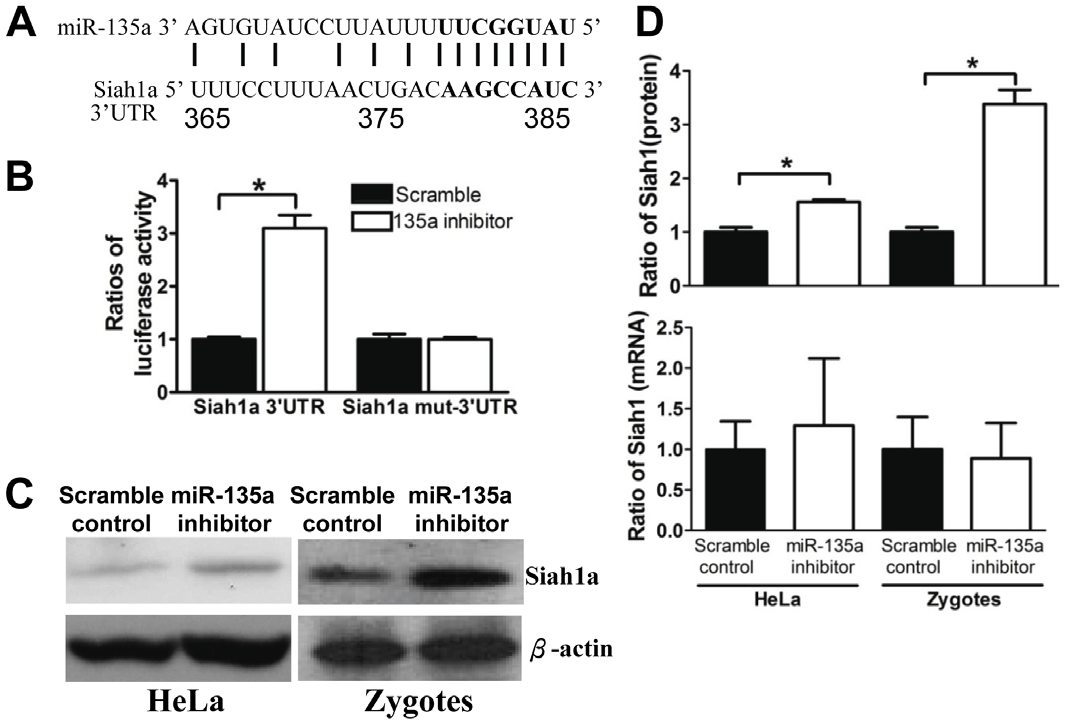
MiR-135a regulates the expression of Siah1. (A) Potential miR-135a binding site on 3′UTR of Siah1a, seed binding region were labeled in bold (position 380–386). (B) Luciferase activity was increased upon transfection of miR-135a inhibitor with Siah1a 3′UTR when compared to the scramble control group. MiR-135a inhibitor did not affect the reporter activity when the Siah1a 3′UTR carried a mutated seed binding region. (C) MiR-135a inhibitor treatment increased the expression of Siah1 in HeLa cells and zygotes. (D) Graphical presentation of means±SD of Siah1 mRNA and protein expressions derived from at least 3 independent experiments. Levels are presented as ratio to the scramble controls. * denotes statistically significant difference between groups (*p*<0.05).

Three publicly accessible miRNA target prediction programs, Pictar, TargetScan 5.2 and miRanda predict that Siah1 is a target of miR-135a. PicTar [32] identified that miR-135a potentially bound to position 365–387 of the 3′UTR of Siah1a (Figure 3A). To verify the *in-silico* prediction; the reporter gene approach [33]–[35] was used. Either miR-135a inhibitor or scramble control was co-transfected with the luciferase reporter gene system, which included a vector carrying the luciferase reporter gene anchoring a Siah1a miR-135a potential binding site.

Transfection of miR-135a inhibitor into HeLa cells suppressed the function of endogenous miR-135a, and thus significantly increased the activity of the luciferase reporter when compared to cells transfected with the scramble control (Figure 3B). When the seed region of the reporter was mutated, miR-135a inhibitor did not affect the luciferase activity (Figure 3B). The expression of Siah1a in miR-135a inhibitor-treated cells and zygotes was also examined. It was found that the Siah1a protein expression was increased in both groups (Figure 3C and D), confirming that miR-135a regulated the expression of Siah1a. The action of miR-135a on Siah1a expression was not by inducing mRNA target degradation as miR-135a inhibitor had no effect on Siah1a mRNA expression in the 2 groups (Figure 3D).

### Localization and expression of Siah1a in oocytes and preimplantation embryos

**Figure 4:**
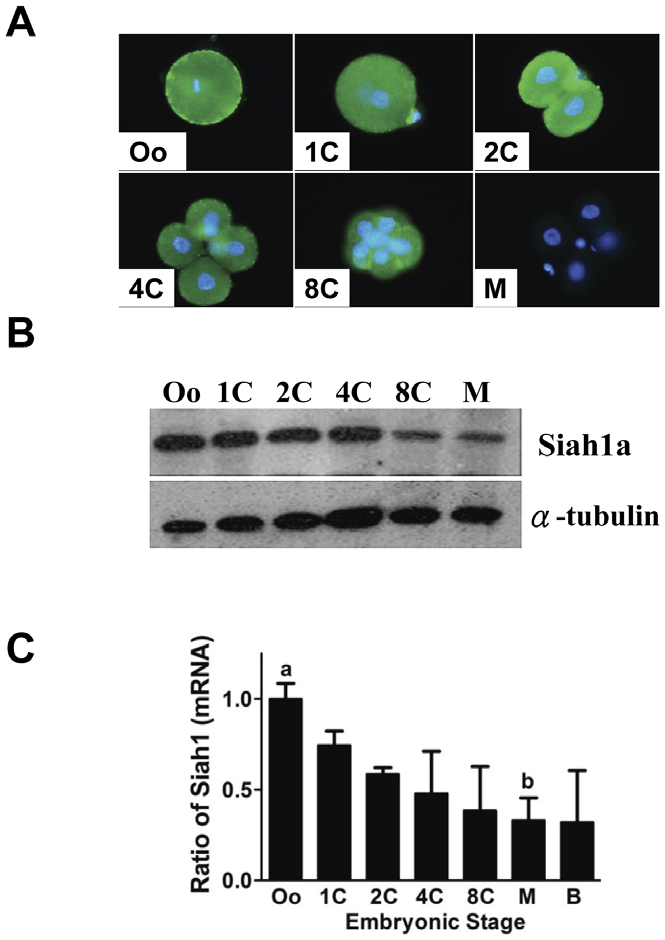
Siah1a protein expression in preimplantation stages embryos. (A) Immunostaining of Siah1a in oocytes and preimplantation stages embryos. (B) Western blotting determination of Siah1a expression in oocytes and preimplantation embryos at different stages of development. (C) Real-time quantification of Siah1a expression in different embryonic stages. Levels are presented as ratio to oocytes (^a,b^ denotes *p*<0.05).

The protein expression of Siah1a during preimplantation embryogenesis was studied by western blotting and immunohistochemical staining. Siah1a immunoreactivities were observed in the cytoplasm of oocytes, zygotes, blastomeres of 2-, 4- and 8-cell embryos (Fig. 4A). Western blot showed that the protein expression of Siah1a decreased continuously with development and noticeable decrease in the levels of Siah1a protein was observed after the 4-cell stage (Figure 4B). Siah1a mRNA also showed a similar trend; the mRNA level was highest in oocytes and decreased upon cleavage (Figure 4C). It is interesting to note that the increased expression of miR-135a in the zygotes correlates with a gradual decrease of Siah1a from this stage (Figure 1 and 4).

### Siah1a mediates the effect of miR-135a on embryo development in mice

**Figure 5:**
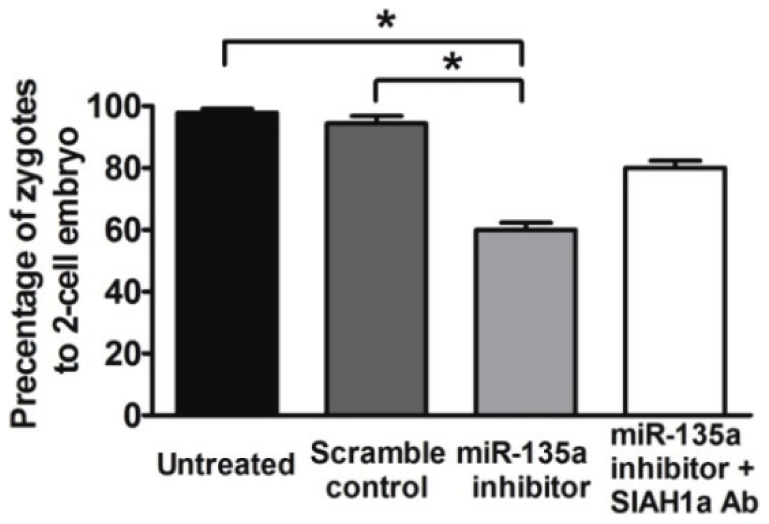
Siah1a antibody partially nullified the effect of miR-135a inhibitor microinjection. Microinjection of Siah1a antibody increased the number of zygotes to 2-cell embryos. The experiment was repeated for at least 3 times and each data point involved at least 50 embryos. * denotes statistically significant difference between groups (*p*<0.05).

We next tested whether Siah1a mediated the inhibitory effect of miR-135a on embryo development. Siah1a specific antibody or water control was co-injected with miR-135a inhibitor or scramble control into pronucleated zygotes. Only about 60% of zygotes injected with miR-135a inhibitor developed to the 2-cell stage. Coinjection with Siah1a antibody partially nullified the effect of miR-135a inhibitor and about 80% of these embryos developed to the 2-cell stage (Figure 5).

### MiR-135a affects proteasomal degradation and expression of Kid

**Figure 6:**
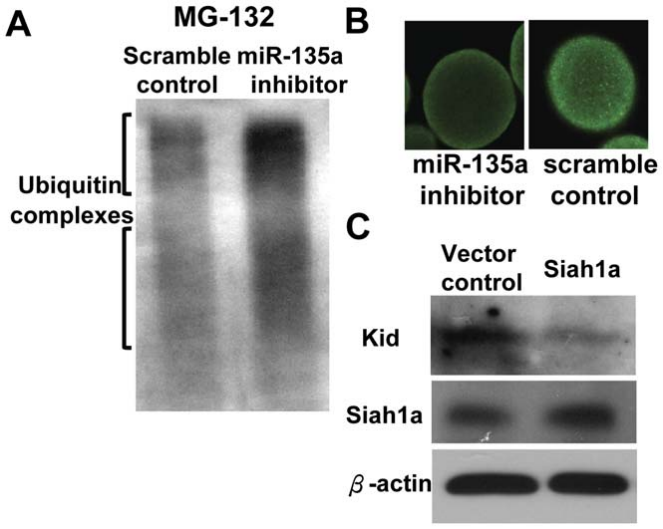
MiR-135a regulates proteasomal degradation. (A) Enhanced accumulation of ubiquitination complex was observed in MG-132 treated miR-135a inhibitor injected zygotes when compared to control embryos. (B) Expression of Kid was decreased in miR-135a inhibitor treated embryos. (C) Forced-expression of Siah1a decreased the level of Kid when compared to vector control in HeLa cells.

Since Siah1a is involved in proteasomal degradation, we examined the action of miR-135a in ubiquitation by using proteasomal inhibitor MG-132. It was found that MG-132 increased the accumulation of ubiquitination complex (Figure 6A). One of the potential Siah1 degradation target is Kid [31]. Therefore, we examined the effect of miR-135a inhibitor on Kid expression. Immunostaining showed that the Kid level was decreased in miR-135a inhibitor treated embryos (Figure 6B). To demonstrate the relationship between Siah1a and Kid expression, we constructed an expression vector of Siah1a. Forced-expression of Siah1a in HeLa cells results in reduced Kid expression (Figure 6C).

## Discussion

Embryonic development is a tightly regulated process. MiRNAs are important regulators fine tuning the expression of many genes simultaneously. They are important in early embryo development of *C. elegans* [5]–[7], *Drosophila* [8]–[10], fish [11] and mammals [36]. Mouse preimplantation embryos expressed developmental stage-specific miRNAs [4], [37], [38] and miR-135a is specifically expressed in the zygotic stage. Here, we demonstrated that miR-135a is important for the first cell cleavage; microinjection of miR-135a inhibitor reduced the percentage of zygotes undergone first cell division by about 30%.

Several observations indicate that the action of miR-135a is partially mediated through Siah1a. First, luciferase reporter assay showed that miR-135a acted on the 3′UTR of Siah1 directly but not on the mutated seed binding region. Second, miR-135a knockdown induced an increase in Siah1 protein in mouse zygotes and HeLa cells. Third, the inhibitory activity of miR-135a knockdown at zygote stage was partially nullified by co-injection of anti-Siah1a antibody. It is not surprising that the antibody cannot completely nullify the effect of miR-135a inhibitor because miRNA regulates a number of target genes simultaneously and the treatment nullifies the effect of Siah1 only.

Knockout models have been used to study the role of Siah1 genes in development. Siah1b is essential for embryonic development because live chimeras are born at a very low frequency, and many of them exhibited abnormalities [29]. Siah1a knockout mice also suffer from severe abnormalities [39]. About 70% of Siah1a^−/−^ pups died in the nursing period, and mature mice have small liver and testis. Siah1a^−/−^ males are sterile while Siah1a^−/−^ females are subfertile. Although Siah2 shares high homology with Siah1 genes, Siah2 knockout mice are fertile and have largely normal phenotype [40]. However, when combined with Siah1a knockout, around 75% of the mice died within 1 hour of birth and nearly none survived by day 5. These observations clearly indicate the importance of Siah genes in development and fertility. Although Siah1a is essential for normal development, our study showed that the level of Siah1a needs to be tightly control at the zygotic stage, as excessive amount of the enzyme induced by miR-135a inhibitor inhibited first cleavage division. The observation is consistent with the idea that miRNAs act as rheostats fine-tuning protein expression [41].

Siah1 is an E3 ubiquitin ligase involving in ubiquitination. Thus, the present data demonstrate that in addition to degradation of the translation of its target gene Siah1, miR-135a also regulates the level of other proteins indirectly by controlling the expression of ubiquitin-specific proteases. Although zygotic genomic activation is crucial to embryo development, degradation of maternal transcripts [42]–[44] and proteins are equally important [45], [46]. Ubiquitination is essential for the dynamic control of protein turnover in embryo development. It has been shown that the E3 ubiquitin ligase, Ret Finger-Like 4 gene (RFLP4) is involved in the ubiquitin proteasome degradation pathway and interacts with target proteins such as cyclin B1 for degradation during oocyte-embryo transition [45]. Another E3 ubiquitin ligase known to be important for embryonic development is G2E3, which acts to prevent apoptosis of early embryos, and its deficiency leads to death of embryos prior to implantation [47]. The importance of ubiquitination is also suggested by the differential expression of ubiquitin proteasome genes in embryos obtained after different hormonal stimulation protocols [48].

In this study, the expression of Siah1a was high in oocytes, and decreased in the zygotic stage when the level of miR-135a was high. The expression pattern is similar to that of RFLP4, which is highly expressed in the mouse oocytes but is much reduced in the 2-cell embryos [45]. The similarity in the expression and function of these E3 ubiquitin ligases tempt us to speculate that down-regulation of these enzymes is important in the first cleavage division. The validity of this hypothesis remains to be tested. The expression levels of Siah1a did not increase after the 4-cell stage when the miR-135a level was low. Thus, other unknown miRNAs or mechanism(s) are inhibiting the transcription and/or translation of the Siah1a gene.

The inhibitory action of miR-135a knockdown on the first cell cleavage could result from perturbation of the proteolysis of Siah1a substrates. Indeed, immunoprecipitation and yeast two-hybrid screening have demonstrated that Siah1 interacts with a number of proteins that are involved in cell cycle arrest or apoptosis, including POSH [49], HIPK2 [50], Numb [51], Pw1/Peg3 [27], transforming growth factor β [52] and BAG-1 [53]. Whether these proteins are interacting with Siah1 in early oocytes-embryo transition needs further investigation.

Kid is one of the Siah1 effector [31]. It is a microtubule-associated motor protein essential for chromosome compaction to ensure proper nuclear envelope formation by reducing anaphase chromosome mass [30]. Mouse deficient of Kid have fragmented pronuclei in zygote and multi-nuclei in blastomere of early embryos; and half of them die prior to E9.5 [30]. Here, we observed that the Kid level was decreased upon miR-135a inhibition; consistent with the reduction in Siah1a expression mediated by zygotic increase of miR-135a enhances the level of Kid in zygotes for normal first cleavage division.

Besides protein ubiquitination, evidence also suggests that Siah1 inhibits cell proliferation and promotes apoptosis [26]–[28]. Ovulated oocytes that are not fertilized dies by apoptosis to prevent the development of abnormal conceptus [54]. The expression of caspase 3 is higher in aged mouse oocytes than in young oocytes [55]. On the other hand, p53 induces Siah1 mediate cell growth arrest though inhibiting DNA synthesis without inducing apoptosis in epithelial and fibroblast cell models [53]. The importance of miR-135a in preventing death of oocyte/zygote remains to be determined.

There are reports showing that the miRNA function is suppressed in mouse oocytes [14], [15]. Our unpublished data show that miRNAs regained their activity after fertilization. Mouse embryos deficient of Dgcr8 produced by heterozygous mating die by E6.5 [16]. Although Dgcr8 null females produced offsprings with wild-type males, their brood size was reduced by about 40%, leading to the conclusion that maternal Dgcr8 affects female fecundity though it is not required for fertility [15]. These reports did not study the role of sperm miRNAs in embryo development. Our unpublished data show that the miRNAs of spermatozoal origin are delivered to the fertilized zygotes after fertilization and affect first cleavage division. Mature miR-135a can be detected in mouse epididymal spermatozoa at a level that is 2.2-fold higher than that of oocytes.

In summary, we have demonstrated that miR-135a is involved in the first cell cleavage in zygotes. The action of miR-135a is, at least partially through down-regulation of Siah1a.

## Materials and Methods

### Mouse embryo collection

The protocol of this study was approved by the Committee on Use of Live Animals in Teaching and Research (approval number: 1531-07), the University of Hong Kong. ICR female mice aged 6–8 weeks were superovulated by consecutive injections of 5 IU of pregnant mare serum gonadotropin (Sigma, St. Louis, MO, USA) and 5 IU of human chorionic gonadotropin (hCG, Sigma) 47–48 hours apart. Unfertilized eggs were harvested from females at 21-hour post-hCG injection without mating. Fertilized zygotes (1-cell embryos) were harvested from the mated mice at 21-hour post-hCG. Embryos at the 2-cell, 4-cell, 8-cell, and morulae stages were flushed out from the oviducts at 40–42, 54–56, 62–64, and 76–78 hour behind hCG injection, respectively. Blastocysts were retrieved from the uterine horns at 86–88 hour post-hCG injection.

### RNA extraction and TaqMan real-time PCR

For miRNA expression analysis, 5 embryos at the same developmental stage were pooled for RNA extraction. Reverse transcription was performed by TaqMan ® MicroRNA Reverse Transcription Kit (Applied Biosystems, Carlsbad, CA). MicroRNA expression was determined with TaqMan® MicroRNA assay (Applied Biosystems) according to the manufacturer's protocol by using the Applied Biosystems 7500 Detection system (Applied Biosystems).

### Microinjection and *in vitro* embryo culture

About 10 pL of 25 µM locked nucleic acid modified miR-135a inhibitor (miRCURY LNA™ microRNA inhibitors, Exiqon, Vedbaek, Denmark) was microinjected into the cytoplasm of the zygotes. Scramble control (miRCURY LNA™ microRNA antisense controls, LNA probe of similar length without homology to any known miRNA or mRNA sequence in human, mouse or rat) injected embryos were used as control for assessing injection damage. After microinjection, groups of 20–30 embryos were cultured in 40 µl of KSOM medium supplemented with amino acids (Chemicon, Billerica, MA) and overlaid with mineral oil at 37°C in an atmosphere of 5% CO_2_ for 4 days. Embryo development was observed under an inverted microscope.

### MicroRNA-135a inhibition and luciferase reporter assay

Oligonucleotides were synthesized according to the sequence of the potential binding regions identified by PicTar [32] (http://pictar.bio.nyu.edu/). Digestion sites for NotI and XhoI were added to the 5′ and 3′ end of each site in the 3′UTR of the predicted target. The sequences of the original and mutated oligonucleotides were listed in Materials and Methods S1. The double digested fragments were cloned downstream of the luciferase gene between the XhoI/NotI site of psiCHECK™-2 vector (Promega, Madison, WI). MiR-135a inhibitor (human miR-135a is identical to mouse miR-135a and the same inhibitor can be used), or scramble control were transfected together with reporter constructs into HeLa cells (American Type Culture Collection, Manassas, VA). Transfection and assay procedures were described as in [56].

### Immunofluorescence staining of embryos

Fresh collected pre-implantation embryos were washed with Dulbecco's PBS (DPBS) containing CaCl_2_ and MgCl_2_ (1 mM each) and fixed in 4% paraformaldehyde for 15 minutes at room temperature. Embryos were permeabilized with 0.1% Triton X-100 in DPBS for 4 minutes, incubated for 1.5 hour in DPBS containing 10% goat serum at room temperature, and reacted with antibody against Siah1a (Abcam, UK) or Kid (Santa Cruz Biotechnology, Inc, Santa Cruz, CA), at 4°C overnight followed by secondary antibody (fluorescein isothiocyanate, FITC-labeled anti-goat IgG) for 1 hour at 37°C. Nuclei were stained with 5 µg/ml DAPI (Sigma) for 5 minutes. Finally, the embryos were rinsed in DPBS to remove excess reagents, and examined under fluorescence microscope.

### Forced-expression of Siah1a and Western blotting

The coding region of Siah1a was cloned into the pTracer™-CMV/Bsd vector. Successful of forced-expression was confirmed by Western blotting using specific anti-Siah1a antibody (Abcam).

For examining the expression of Siah1a in preimplantation embryos, oocytes and embryos at different stages of development were collected, rinsed twice in PBS, resuspended in 10 µl of Laemmli buffer and boiled for 10 minutes. The proteins were resolved on SDS-PAGE as described [56], each lane represented a pool of 40 embryos. Specific anti-Siah1a antibody (Abcam) was used to detect Siah1a expression. For assessing Siah1a expression upon miR-135a knockdown, miR-135a inhibitor was microinjected into zygotes or transfected into HeLa cells. Scramble control treated zygotes or HeLa cells were used as control.

### MG-132 inhibition

Immediately after microinjection, the zygotes were incubated in 5 µM of MG-132 (Sigma) to inhibit proteasomal degradation. The embryos were collected 6 hours later and subjected for Western blotting. Ubiquitin level was assessed by ubiquitin specific antibody (Abcam).

### Data Analysis

All the results are shown as means ± Standard deviation (SD). All the data were analyzed using one-way analysis of variance (ANOVA). A *p*-value of less than 0.05 was considered to be statistical significance.

## Supporting Information

Materials and Methods S1 **Oligonucleotides used for generating the Siah1a 3′UTR luciferase reporter.**
(DOC)
