# Supplementary material for: miR-135A Regulates Preimplantation Embryo Development through Down-Regulation of E3 Ubiquitin Ligase Seven in Absentia Homolog 1A (SIAH1A) Expression
Source: PLoS One. 2011 Nov 22;6(11):e27878. doi: 10.1371/journal.pone.0027878 (PMC3222661; doi:10.1371/journal.pone.0027878)
Supplement: Materials and Methods S1 — Oligonucleotides used for generating the Siah1a 3′UTR luciferase reporter. (DOC) [file pone.0027878.s001.doc]

**Materials and Methods S1**

**Oligonucleotides used for generating the Siah1a 3’UTR luciferase reporter:**

Siah1a-F: 5’tcgagTTTCCTTTAACTGAC**AAGCCAT**CagTTTCCTTTAACTGAC**AAGCCAT**Cgc

Saih1a-R

5’ ggccgcGATGGCTTGTCAGTTAAAGGAAActGATGGCTTGTCAGTTAAAGGAAAc

Siah1a-mutant-F

5’ tcgagTTTCCTTTAACTGACAA*CACATC*agTTTCCTTTAACTGACAA*CACATC*gc

Saih1a-mutant-R

5’ ggccgcGATGTGTTGTCAGTTAAAGGAAActGATGTGTTGTCAGTTAAAGGAAAc

Oligonucleotides were designed for the potential miR-135a binding site of Siah1a. Digestion sites for NotI/XhoI were added to the 5’ and 3’ end of the oligonucletotide respectively. The potential binding sites for miRNA seed sequence were in bolded while the mutant sites were labeled in italics. To enhance assay sensitivity, each fragment carries 2 copies of the potential binding sequence. After annealing, the double stranded fragment was cloned directly into the pSiCHECK^TM^-2 for luciferase reporter assay.
